# **Multimodality** **assessment of aortic valve area in aortic stenosis: a multicenter validation study**

**DOI:** 10.1007/s10554-025-03576-7

**Published:** 2025-12-01

**Authors:** Christian Weber, J. Studier-Fischer, H. Reiss, S. von Garlen, S. Piepenburg, C. Ehlert, A. Maier, S. Vögele, M. Hein, P. Ruile, J. Fingerhut, S. Jäck, M. T. Hagar, J. Taron, C. Schlett, M. Potratz, T. Rudolph, J. Steffen, D. Hering, S. Deseive, S. Massberg, A. Schwab, J. Leberzammer, P. C. Seppelt, J. Rilinger, M. Zehender, I. Hilgendorf, D. Westermann, C. von zur Mühlen, T. Heidt

**Affiliations:** 1 Department of Cardiology and Angiology, Faculty of Medicine Freiburg, University Heart Center Freiburg - Bad Krozingen, University of Freiburg, Freiburg, Germany; 2 Department of Diagnostic and Interventional Radiology, Faculty of Medicine Freiburg, University of Freiburg, Freiburg, Germany; 3 Clinic for Common and Interventional Cardiology, Heart- and Diabetes- Center North Rhine-Westphalia, Ruhr-University Bochum, Bad Oeynhausen, Germany; 4 Department of Medicine I, LMU University Hospital, Ludwig-Maximilians- University Munich, Munich, Germany; 5 Deutsches Zentrum für Herzkreislaufforschung DZHK e.V, Munich Heart Alliance MHA, Munich, Germany; 6 Department of Cardiology and Angiology, Heart Center University Hospital Frankfurt, Goethe University Frankfurt am Main, Frankfurt am Main, Germany; 7 Division of Cardiology, Max-Grundig Clinics, Buehl, Germany

**Keywords:** Aortic stenosis, Aortic valve area, Computed tomography angiography, Echocardiography, Transcatheter aortic valve replacement

## Abstract

**Objectives:**

Transthoracic echocardiography (TTE) is the standard modality for grading aortic stenosis (AS) severity. Transesophageal echocardiography (TOE) allows direct aortic valve area (AVA) planimetry (AVA_TOE_), while computed tomography angiography (CTA) offers a non-invasive alternative (AVA_CTA_). This study aimed to evaluate the correlation between AVA measurements across modalities and to determine a diagnostic AVA_CTA_ threshold for severe AS.

**Methods:**

This retrospective study included a single-center derivation cohort of 176 patients (mean age 80.0 ± 7.7 years, 52.8% male) with moderate to severe AS who underwent full-cycle CTA, TTE, and TOE. AVA_CTA_ was measured by two independent raters. Correlation with AVA_TOE_ and other parameters was assessed. Receiver operating characteristic (ROC) analysis was used to define an optimal AVA_CTA_ threshold for severe AS, which was validated in a multi-center cohort of 407 patients (mean age 80.9 ± 6.7 years, 52.8% male) with comparable characteristics.

**Results:**

Mean AVA_CTA_ was 0.96 ± 0.28 cm² with a high interrater reliability (IRR = 0.84), compared to a mean AVA_TOE_ of 0.88 ± 0.26 cm² (Pearson’s *r* = 0.73). ROC analysis identified 0.96 cm² as the optimal AVA_CTA_ threshold for diagnosing severe AS (AUC = 0.846; sensitivity = 71.7%; specificity = 89.8%) compared to TOE grading. This threshold yielded good diagnostic performance in the validation cohort (AUC = 0.817; sensitivity = 78.2%; specificity = 72.6%).

**Conclusions:**

AVA_CTA_ demonstrated high reliability, showing a strong correlation with AVA_TOE_. The 0.96 cm² threshold, defined in the derivation cohort, performed well in the validation cohort for assessing aortic stenosis severity.

**Supplementary Information:**

The online version contains supplementary material available at 10.1007/s10554-025-03576-7.

## Introduction

Valvular heart disease, particularly severe aortic stenosis (AS), is the most common valvular disorder in developed countries and remains associated with significant morbidity and mortality [1–3]. Transcatheter aortic valve replacement (TAVR) has become the preferred treatment for many patients over 65 years with severe aortic stenosis, particularly those at intermediate or high surgical risk [4]. Accurate assessment of AS severity is essential and primarily based on transthoracic echocardiography (TTE). According to current guidelines, severe AS is defined by an aortic valve area (AVA) < 1.00 cm² calculated via the continuity equation (AVA_TTE_), a mean transvalvular pressure gradient (AV p_mean_) >40 mmHg, or a peak aortic jet velocity >4.0 m/s [5].

However, low-gradient AS and discordant echocardiographic findings complicate severity assessment [6, 7]. In such cases, computed tomography angiography (CTA) measurement of aortic valve calcification (AVC) is recommended as an adjunct tool in both American and European guidelines [5, 8]. AVC strongly predicts prognosis and features sex-specific cut-offs for severe AS [9]. Moreover, cardiac CTA enables multiplanar reconstruction and direct planimetry of the AVA (AVA_CTA_), providing a potential alternative to transesophageal echocardiography (TOE), the reference method for direct planimetry (AVA_TOE_). While AVA_CTA_ is not yet routinely used clinically, it offers valuable complementary information, especially in anatomically complex or inconclusive cases.

Despite its promise, the clinical role of AVA_CTA_ remains unclear. The optimal strategy to address discordant findings among AVA_TTE_, AVA_TOE_, AVA_CTA_, AVC, and AV pmean is still debated [10–12]. Although AVA_CTA_ and AVA_TOE_ represent anatomical AVA and tend to be larger than effective AVA_TTE_ (due to flow contraction effects), standardized AVA_CTA_ thresholds for grading are lacking [13, 14]. Furthermore, large-scale studies integrating multimodal AS assessment are scarce.

This retrospective multicenter study addresses these gaps by evaluating AVA_CTA_ in a large cohort of patients with moderate to severe AS. We aimed to define an AVA_CTA_ cut-off for severe AS classification in a derivation cohort and validate its diagnostic performance in an independent multicenter cohort. These results may assist clinical decision-making in challenging or discordant cases and clarify AVA_CTA_’s role in multiparametric AS evaluation.

## Methods

### Study cohorts

**Fig. 1:**
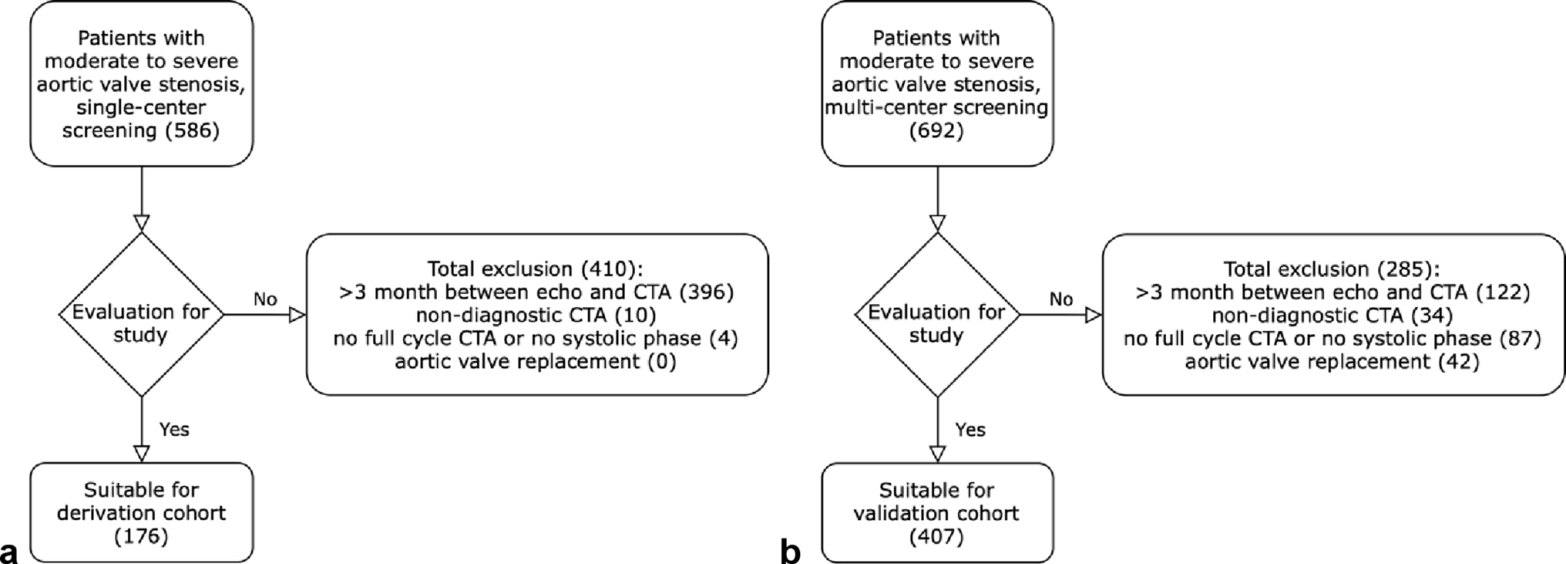
Study flow diagram for the derivation cohort (**a**, left) and the validation cohort (**b**, right)

This study was approved by the ethics commission at the University of Freiburg (vote number 21-1178). It utilized hospital-based data from the University Heart Center Freiburg – Bad Krozingen, Freiburg site (Albert-Ludwig University, Freiburg, Germany), collected between 2014 and 2021, sourced from the Diagnosis Related Groups (DRG) system. As a derivation cohort, adult patients with a diagnosis of symptomatic moderate to severe aortic stenosis (ICD code I35.-) who had undergone all three imaging modalities – TTE, TOE, and CTA – were retrospectively screened and included. Patients were excluded in the presence of bicuspid aortic valves, a history of aortic valve replacement or reconstruction, non-diagnostic CTA, non-systolic phase imaging, incomplete cardiac cycle CTA acquisition, or a time interval of more than three months between the echocardiograms and the CTA. Figure 1a shows the study flow diagram for the derivation cohort.

For the multi-center validation cohort, equivalent patients were retrospectively included from Frankfurt University Hospital (Goethe University, Frankfurt am Main, Germany), LMU Hospital Munich (Ludwig-Maximilians-University, Munich, Germany), University Heart Center Freiburg – Bad Krozingen, Bad Krozingen site (Albert-Ludwig University, Freiburg, Germany), and Heart and Diabetes Center North Rhine-Westphalia (Ruhr-University Bochum, Bad Oeynhausen, Germany) between 2014 and 2023. Figure 1b illustrates the flow diagram for the multi-center enrolled validation cohort. Study participation was approved by the respective ethics committees at each institution (vote numbers 296/16, 2021 − 793_2, 21-1178, 19–840).

## Data protection

Patient data were anonymized prior to analysis to ensure confidentiality and compliance with applicable data protection regulations.

## Echocardiography protocol

All echocardiograms were performed in accordance with current recommendations from the American Society of Echocardiography [5, 15, 16]. At the Freiburg site, Vivid E90 (GE Healthcare, Chicago, IL, USA) and EPIQ CVx (Koninklijke Philips N.V., Amsterdam, Netherlands) ultrasound systems were used. Other centers used comparable equipment routinely available in clinical practice.

## Aortic valve analysis by echocardiography

All echocardiograms were performed by multiple investigators blinded to all CTA imaging data. AVA_TTE_ was calculated using the recorded values for the continuity equation. For patients with atrial fibrillation, at least five spectral Doppler curves were used to evaluate the gradient and velocity. A separate investigator classified AS severity and subtype according to the American Society of Echocardiography guidelines [16]. For the purposes of this study, an AV p_mean_ >40.0 mmHg was considered severe high-gradient AS, with AVA_TTE_ defining further subdivision into severe, concordant (AVA_TTE_ < 1.00 cm²) and severe, discordant (AVA_TTE_ >1.00 cm²) high-gradient AS. The remaining severe (AVA_TTE_ < 1.00 cm²), low-gradient cases were then classified by flow status. Low-flow condition was assumed for stroke volume index (SVi < 35 ml/m²) and subdivided into severe, low-flow, low-gradient (LF/LG) AS with preserved ejection fraction (>50%) and severe LF/LG AS with reduced ejection fraction (< 50%). Severe, normal-flow, low-gradient AS was classified at AV p_mean_ < 40 mmHg, AVA_TTE_ < 1.00 cm², and SVi >35 ml/m². All remaining cases were classified as moderate AS. AVA_TOE_ was evaluated from the clinical records at all hospitals. Planimetry was performed using multiplanar 3D reconstruction, and, as in routine clinical practice, an AVA_TOE_ of less than 1.00 cm² was considered indicative of severe AS based on TOE assessment.

## Computed tomography protocol

For the derivation cohort, CTA scans were performed on a 2nd generation, 128-slices dual-source scanner SOMATOM FLASH (Siemens Healthineers, Forchheim, Germany). The acquisition followed the Society of Cardiovascular Computed Tomography consensus document. The protocol consisted of an unenhanced, prospective electrocardiogram-triggered, high-pitch spiral coronary calcium scoring scan followed by a contrast-enhanced electrocardiogram-gated, retrospective, low-pitch spiral cardiac CTA. 70 ml Imeron 400 (Bracco S.p.A, Milano, Italy) followed by a mixture of 30 ml Imeron 400 and 40 ml isotonic saline at a flow rate of 5 ml/s were administered by a dual-syringe power injector. Bolus tracking initiated the start of cardiac CTA using the left atrium as region of interest. Scanning parameters for cardiac CTA included a tube voltage of 120 kV, automatic tube current selection (Care Dose mAs) and a collimation of 2 × 64 × 0.6 mm. Images were reconstructed at an axial section thickness of 0.6 mm with an increment of 0.6 mm, matrix size was set at 512 × 512 pixels, and a field of view of 180 × 180 mm was applied. Multiphase data was reconstructed automatically at intervals of at least 50 ms between 100 ms and 400 ms of the R-R interval. Vascular convolution kernel (B26f) and iterative reconstruction (ADMIRE 3) were used for all images. CTA acquisition for the other enrolling centers followed each heart center´s specific standard procedure using local CT scanners available.

### Computed tomography valve analysis

**Fig. 2:**
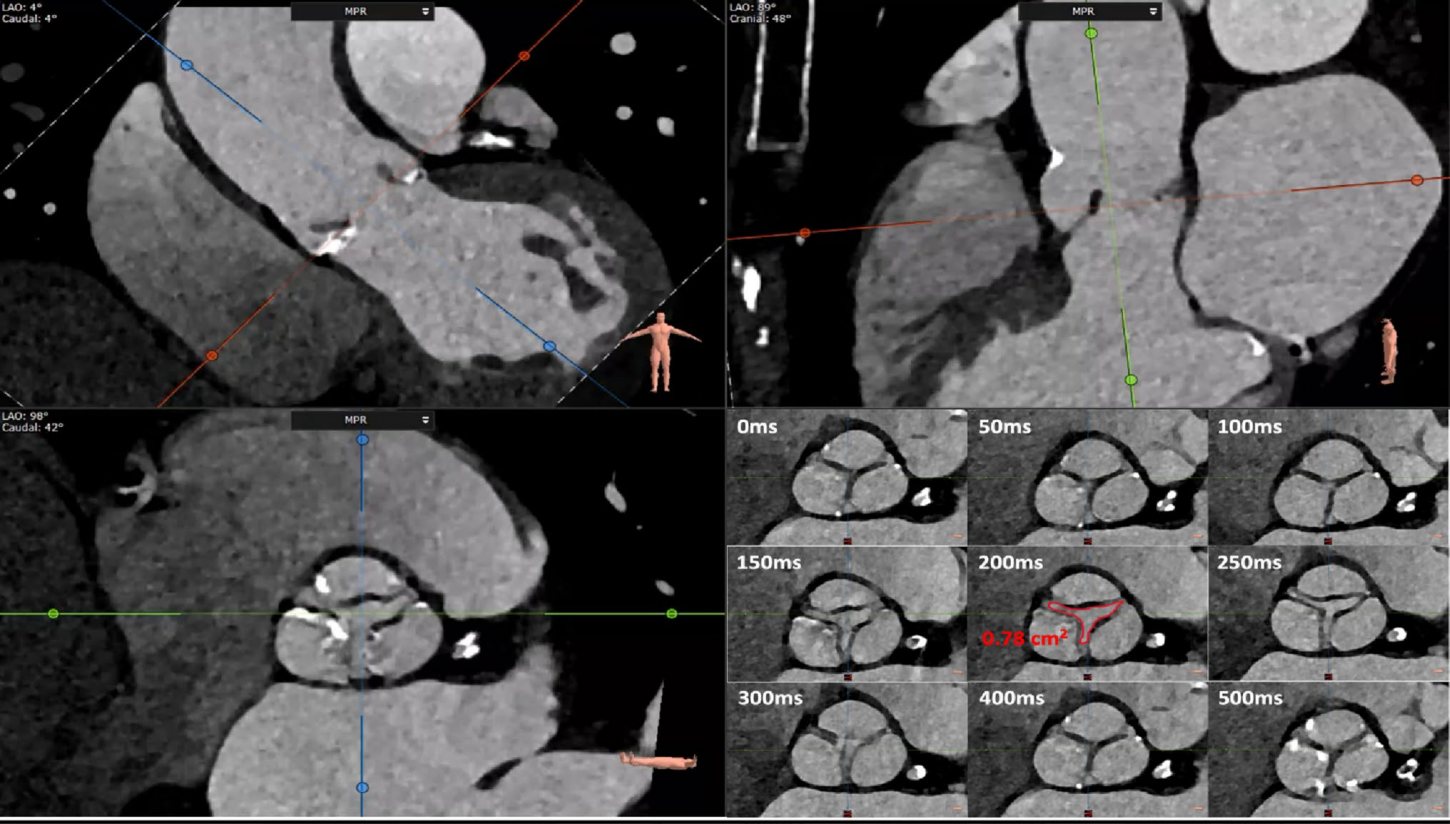
Computed tomography valve analysis. Representative images of multiplanar reconstruction (top left and top right) and visualization of the aortic valve orifice (bottom left). Midsystolic planimetry of the aortic valve area is shown in the bottom right image. White numbers indicate the time (in milliseconds) between the R-wave and image acquisition. The red area represents the midsystolic aortic valve area at 200 ms

All analysis was performed using 3mensio imaging software (3mensio, Maastricht, Netherlands). For the derivation cohort, all CTA scans were reviewed regarding AVC and AVA_CTA_ by two independent investigators blinded to all other imaging and clinical data. AVC was assessed on an unenhanced CT chest scan using the Agatston method in 3 mm axial slices. Calcium associated with other structures of the heart such as coronary arteries and other valves was excluded from the total AVC count. Sex-specific thresholds were used to determine severity regarding the Agatston score (likely severe AS >1200 Agatston units (AU) in women and >2000 AU in men as recommended by the 2021 guidelines of the European Society of Cardiology for the management of valvular heart disease [8]. AVA_CTA_ was measured as described previously [17]. Briefly, maximum aortic valve opening was assessed during a midsystolic phase. By using three orthogonal planes from multiplanar reconstruction, a true short axis view of the aortic valve orifice was measured by minimizing the opening area along the outflow direction. Direct planimetry was performed at the most stenotic level within the aortic valve using the inner edges of the leaflets to obtain minimal AVA_CTA_ during a mid-systolic phase as shown in Fig. 2. For the validation cohort, all comparable CTA scans were reviewed by a single independent investigator blinded to all other imaging and clinical data. AVC and AVA_CTA_ were measured as described above.

### Statistical analysis

All analyses, figures, and tables were performed and created using SPSS version 29.0 (IBM SPSS Statistics, Armonk, USA). Descriptive statistics are presented as counts and percentages for categorical variables and mean ± standard deviation (SD) for continuous variables. Comparisons between patient characteristics were performed using t-tests for continuous variables and χ²-tests for categorical variables. Statistical significance was defined as two-sided *p* < 0.05. Interrater reliability for AVA_CTA_ was assessed within the derivation cohort. Pearson’s correlation coefficients were calculated for AVA_TTE_, AVA_TOE_, AVA_CTA_, AVC, and AV p_mean_. Diagnostic accuracy was evaluated by Receiver Operating Characteristic (ROC) curve analysis. Bland-Altman plots were generated to assess agreement. Optimal thresholds and safety cut-off values for exclusion and confirmation of severe aortic stenosis were determined in the derivation cohort using the Youden index from ROC analyses. These thresholds were subsequently validated in the independent validation cohort.

## Results

### Derivation cohort characteristics

A total of 586 patients with moderate to severe aortic stenosis (AS) meeting the inclusion criteria were screened at the University Heart Center Freiburg - Bad Krozingen, Freiburg site. Overall, 410 patients were excluded: 396 due to the interval between TTE, TOE, and CTA acquisitions exceeding three months, 10 due to poor CTA image quality, and 4 due to non-systolic phase or incomplete CTA acquisition. Finally, 176 patients were included (131 severe AS [74.4%] and 45 moderate AS [25.6%] by TTE classification). The study flow diagram for the derivation cohort is shown in Fig. 1a. Baseline characteristics are summarized in Table 1.

**Table 1:** Patient characteristics of the derivation and validation cohort:

	Derivation cohort	Validation cohort	*p*-value
N	176	407	
Age, years	80.0 ± 7.7	80.9 ± 6.7	0.166
**Gender** Male, n (%) Female, n (%)	93 (52.8) 83 (47.2)	215 (52.8) 192 (47.2)	0.997 0.997
**Body measurements** Height, cm Weight, kg BMI, kg/m^2^	167.8 ± 9.4 77.3 ± 16.4 27.0 ± 4.7	168.0 ± 8.9 76.3 ± 15.7 27.0 ± 4.7	0.865 0.473 0.861
**TTE findings** LVEF, % >50, n (%) 30–50, n (%) < 30, n (%) AV p_mean_, mmHg AVA_TTE_, cm^2^ Diastolic dysfunction, n (%) Stroke volume, ml/m^2^	50.5 ± 11.5 117 (66.5) 44 (25.0) 15 (8.5) 33.1 ± 12.7 0.86 ± 0.23 97 (55.1) 39.2 ± 10.8	49.9 ± 10.6 262 (64.4) 111 (27.3) 34 (8.4) 33.3 ± 13.1 0.84 ± 0.22 110 (27.0) 37.0 ± 11.7	0.587 0.908 0.339 < 0.001 ** 0.042 *
**TOE findings** AVA_TOE_, cm^2^	0.88 ± 0.23	0.83 ± 0.24	0.042 *
**CT findings** AV calcium score, AU AVA_CTA_, cm^2^	2659.6 ± 1796.2 0.96 ± 0.24	1968.8 ± 1908.4 0.88 ± 0.22	< 0.001 ** < 0.001 **
**Preexisting illnesses** Diabetes mellitus, n (%) Hypertension, n (%) Nicotine abuse, n (%) CAD, n (%) Atrial fibrillation, n (%)	66 (37.5) 147 (83.5) 42 (23.9) 118 (67.0) 100 (56.8)	133 (32.7) 333 (81.8) 73 (17.9) 247 (60.7) 126 (31.0)	0.260 0.621 0.099 0.146 < 0.001 **

The mean age was 80.0 ± 7.7 years, with 93 men (52.8%) and 83 women (47.2%). Mean height was 167.8 ± 9.4 cm, weight 77.3 ± 16.4 kg, and BMI 27.0 ± 4.7 kg/m². Echocardiography revealed a mean AVA_TTE_ of 0.86 ± 0.23 cm² and a mean AV p_mean_ of 33.1 ± 12.7 mmHg. Mean AVA_TOE_ was 0.88 ± 0.23 cm². According to AS subgroups, 40 patients (22.7%) had concordant high-gradient AS, 2 (1.1%) discordant high-gradient AS, 30 (17.0%) low-flow/low-gradient (LF/LG) AS with reduced ejection fraction (EF), 16 (9.1%) LF/LG AS with preserved EF, 43 (24.4%) normal-flow low-gradient AS, and 45 (25.6%) moderate AS. Notably, 59 patients (33.5%) had a reduced stroke volume index (SVi < 35 ml/m²) indicative of low-flow condition.

CT analysis revealed a mean AVC of 2659.6 ± 1796.2 AU, with 122 patients (69.3%) exceeding sex-specific thresholds for severe AS. Mean AVA_CTA_ was 0.96 ± 0.24 cm², measured by two independent investigators with good interrater reliability (IRR = 0.84). When comparing AVC to AVA_TTE_, 82.0% (100/122) of patients classified as severe by AVC showed AVA_TTE_ < 1.00 cm², whereas only 42.6% (23/54) of patients with non-severe AVC also displayed non-severe AS with AVA_TTE_ >1.00 cm².

### Diagnostic performance and correlation of AVA_CTA_

**Fig. 3:**
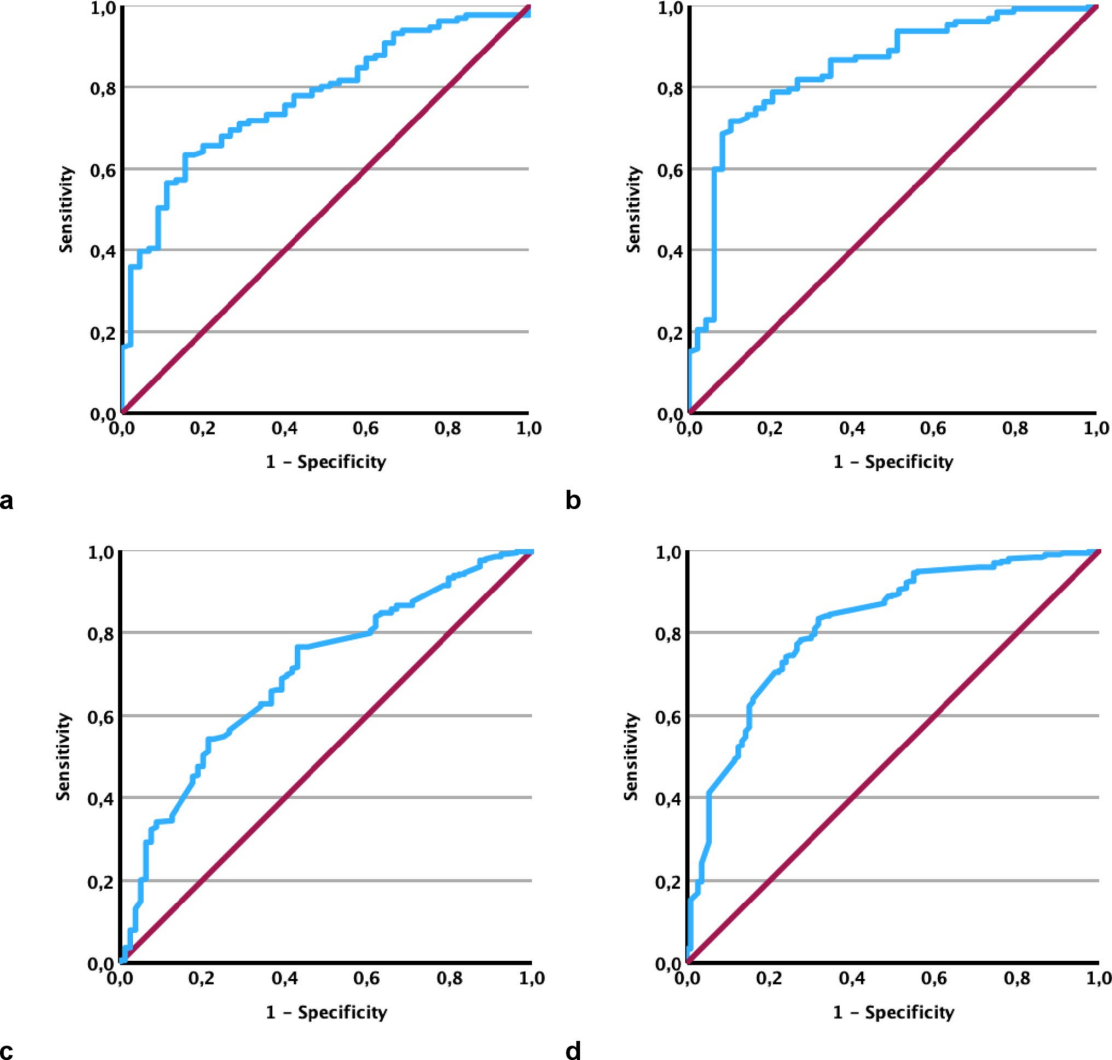
Receiver operating characteristic (ROC) curves for classification of aortic stenosis severity using AVA_CTA_ compared with reference modalities. Left: Comparison with AVA_TTE_; right: comparison with AVA_TOE_. Panels **a** and **b** show results for the derivation cohort; panels **c** and **d** show results for the validation cohort. ROC, receiver operating characteristic; AVA, aortic valve area; AVACTA, AVA measured by computed tomography planimetry; AVATTE, AVA calculated by continuity equation in transthoracic echocardiography; AVATOE, AVA measured by transesophageal echocardiography planimetry

**Fig. 4:**
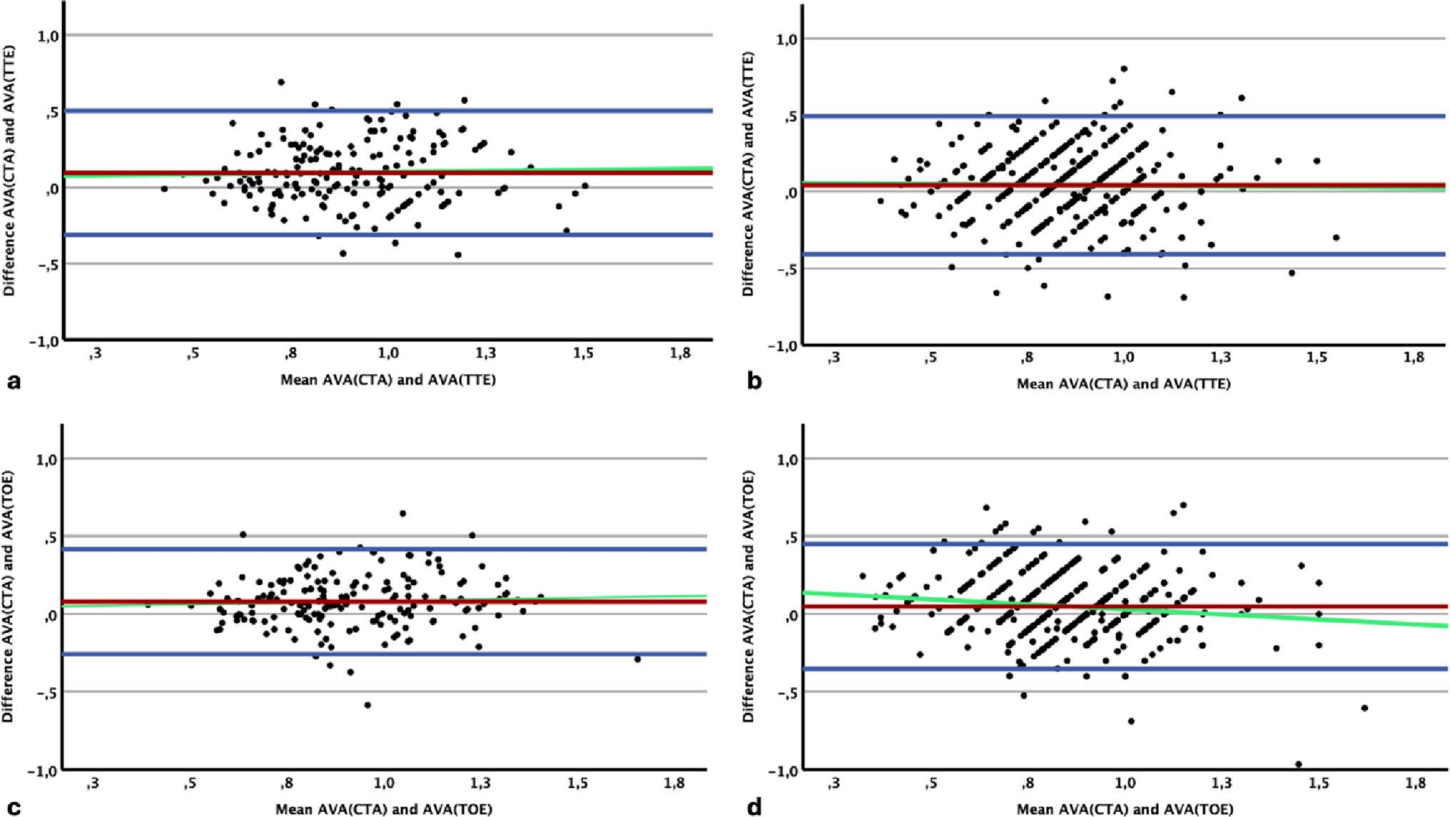
Bland-Altman-plots of agreement of TTE, TOE and CTA measurements of AVA. Agreement between AVA_CTA_ and AVA_TTE_ in the derivation (**a**) and validation (**b**) cohorts; agreement between AVA_CTA_ and AVA_TOE_ in the derivation (**c**) and validation (**d**) cohorts. The red line indicates the mean difference; blue lines represent mean difference ± 1.96 standard deviations (SD); green line shows linear regression of the mean and the difference. All values are in cm². AVA, aortic valve area; AVACTA, AVA measured by computed tomography planimetry; AVATTE, AVA calculated by continuity equation in transthoracic echocardiography; AVATOE, AVA measured by transesophageal echocardiography planimetry; TTE, transthoracic echocardiography; TOE, transesophageal echocardiography; CT, computed tomography; CTA, computed tomography angiography; SD, standard deviation

Receiver operating characteristic (ROC) analyses and Bland-Altman plots evaluated the diagnostic performance of AVA_CTA_ against AVA_TTE_ and AVA_TOE_ classifications, demonstrating good agreement and small absolute differences (Figs. 3 and 4; Table 2**/3**). The area under the curve (AUC) of AVA_CTA_ compared to TOE classification was 0.846. Using AVA_CTA_ safety cut-off values of 1.17 cm² (for exclusion) and 0.95 cm² (for confirmation), sensitivity and specificity exceeded 90% (90.6% and 91.8%, respectively). A single best cut-off value of 0.96 cm² resulted in 71.7% sensitivity and 89.8% specificity. For TTE classification, the AUC was 0.774, with cut-offs of 1.22 cm² (90.1% sensitivity) and 0.88 cm² (91.1% specificity), and a single cut-off of 0.95 cm² yielding 63.4% sensitivity and 84.4% specificity.

**Table 2:** Diagnostic performance of AVA_CTA_ compared to TTE classification in the derivation and validation cohorts.:

Threshold	*N*	AUC	Accuracy (%)	Sensitivity (%)	Specificity (%)	PPV (%)	NPV (%)
AVA_CTA_ threshold 1.22 cm^2^ Derivation cohort Validation cohort	176 407	0.774 0.698	76.1 80.9	90.1 97.3	35.6 12.7	80.3 82.2	55.2 52.9
AVA_CTA_ threshold 0.95 cm^2^ Derivation cohort Validation cohort	176 407	0.774 0.698	68.8 67.3	63.4 68.9	84.4 60.8	92.2 87.9	44.2 32.0
**AVA**_**CTA**_ **threshold 0.88 cm**^**2**^ Derivation cohort Validation cohort	176 407	0.774 0.698	60.8 58.7	50.4 54.6	91.1 75.9	94.3 90.4	38.7 28.7

**Table 3:** Diagnostic performance of AVA_CTA_ compared to TOE classification in the derivation and validation cohorts.:

Threshold	*N*	AUC	Accuracy (%)	Sensitivity (%)	Specificity (%)	PPV (%)	NPV (%)
AVA_CTA_ threshold 1.17 cm^2^ Derivation cohort Validation cohort	176 407	0.846 0.817	79.0 76.7	90.6 97.3	49.0 23.0	82.2 76.7	66.9 76.7
AVA_CTA_ threshold 0.96 cm^2^ Derivation cohort Validation cohort	176 407	0.846 0.817	76.8 76.6	71.7 78.2	89.8 72.6	94.8 88.1	55.1 56.2
**AVA**_**CTA**_ **threshold 0.95 cm**^**2**^ Derivation cohort Validation cohort	176 407	0.846 0.817	75.0 76.4	68.5 77.9	91.8 72.6	95.6 88.1	52.9 55.8

Pearson correlation coefficients (Table 4) showed that AVA_CTA_ correlated strongest with AVA_TOE_ (*r* = 0.73), slightly better than AVA_TTE_ and AVA_TOE_ correlation (*r* = 0.62). Mean gradient (AV p_mean_) showed a modest negative correlation with AVA_CTA_ (*r* = -0.33) and AVA_TTE_ (*r* = -0.31). AVC exhibited the lowest correlations of all combinations depicting almost no relationship to any investigated parameter.

**Table 4:** Correlation of aortic valve parameters:

	Cohort	AVA_TTE_	AVA_TOE_	AVA_CTA_	AVC	AV *p*_mean_
**AVA**_**TTE**_	Derivation cohort Validation cohort	1 - 1 -	0.63 < 0.001 ** 0.48 < 0.001 **	0.61 < 0.001 ** 0.44 < 0.001 **	-0.06 0.417 0.02 0.798	-0.31 < 0.001 ** -0.26 < 0.001 **
**AVA**_**TOE**_	Derivation cohort Validation cohort	0.63 < 0.001 ** 0.48 < 0.001 **	1 - 1 -	0.73 0.001 ** 0.60 0.001 **	-0.11 0.138 0.23 < 0.001 **	-0.41 < 0.001 ** -0.24 < 0.001 **
**AVA**_**CTA**_	Derivation cohort Validation cohort	0.61 < 0.001 ** 0.44 < 0.001 **	0.73 0.001 ** 0.60 0.001 **	1 - 1 -	-0.09 0.226 0.10 0.133	-0.33 < 0.001 ** -0.23 < 0.001 **
**AVC**	Derivation cohort Validation cohort	-0.06 0.417 0.02 0.798	-0.11 0.138 0.23 < 0.001 **	-0.09 0.226 0.10 0.133	1 - 1 -	0.38 < 0.001 ** 0.22 0.001 *
**AV p**_**mean**_	Derivation cohort Validation cohort	-0.31 < 0.001 ** -0.26 < 0.001 **	-0.41 < 0.001 ** -0.24 < 0.001 **	-0.33 < 0.001 ** -0.23 < 0.001 **	0.38 < 0.001 ** 0.22 0.001 *	1 - 1 -

### Validation cohort characteristics

A total of 692 patients meeting inclusion criteria were identified across four heart centers. Of these, 285 were excluded due to > 3 months between echocardiography and CTA (*n* = 122), non-diagnostic CTA (*n* = 34), non-systolic or incomplete CTA (*n* = 87), or prior aortic valve replacement (*n* = 42). The validation cohort comprised 407 patients, with 328 (80.6%) classified as severe AS and 79 (19.4%) as moderate AS by TTE (Fig. 1b). Baseline characteristics showed no significant differences except for some parameters detailed in Table 1.

Mean AVA_TOE_ and AVA_CTA_ were slightly smaller at 0.83 ± 0.24 cm² and 0.88 ± 0.22 cm², respectively. Diastolic dysfunction and atrial fibrillation were less frequent in the validation cohort. Stroke volume index was available for 350 patients (86.0%), with 170 (41.8%) showing SVi < 35 ml/m² indicating low-flow status. The AS subgroup distribution of the combined cohorts is presented in **Supplementary Table 1**.

AV calcium score was available in 214 patients (52.6%), with 90 (43.9%) exceeding sex-specific thresholds. Mean AVC was 1968.8 ± 1908.4 AU, confirming severe calcification in both cohorts. When comparing AVC to AVA_TTE_, 71.1% (64/90) of severe AVC patients had AVA_TTE_ < 1.00 cm², while 89.5% (111/124) of non-severe AVC patients also had AVA_TTE_ >1.00 cm².

### AVA_CTA_ validation of diagnostic performance

ROC and Bland-Altman analyses confirmed the diagnostic performance of AVA_CTA_ in the validation cohort (Figs. 3 and 4; Table 2**/3**). The AUC was 0.817 for TOE and 0.698 for TTE classification. Using derivation cohort cut-offs (upper: 1.17 cm²; lower: 0.95 cm²) against TOE classification yielded 97.3% sensitivity for exclusion and 72.6% specificity for confirmation of severe AS. The single best cut-off (0.96 cm²) achieved 78.2% sensitivity and 72.6% specificity. For TTE classification, cut-offs (1.22 cm² and 0.88 cm²) yielded 97.3% sensitivity and 75.9% specificity; the single cut-off (0.95 cm²) showed 68.9% sensitivity and 60.8% specificity.

Correlation coefficients in the validation cohort (Table 4) were slightly lower but comparable to the derivation cohort. AVA_CTA_ showed moderate correlation and best agreement with AVA_TOE_ (*r* = 0.60), while AVC again showed the lowest correlations with other parameters.

## Discussion

This is the first study to analyze two retrospective cohorts of patients with moderate and severe AS, all of whom underwent comprehensive TAVR evaluation using TTE, TOE, and CTA for AVA assessment. Uniquely, it directly compares all three modalities and validates the findings of the derivation cohort within an independent, multi-center enrolled validation cohort.

While AVA_TTE_ reflects the effective orifice area, AVA_TOE_ and AVA_CTA_ are intended to represent larger, anatomically derived areas [13, 14]. In our cohorts, however, AVA_TTE_ and AVA_TOE_ yielded comparable measurements, which may be attributed to bias introduced by unblinded TOE following TTE. As expected, AVA_CTA_ produced slightly larger values (ΔAVA_meanCTA–meanTOE_ = 0.05 cm²; ΔAVA_meanCTA–meanTTE_ = 0.05 cm²; ΔAVA_meanTOE–meanTTE_ = 0.00 cm²).

ROC analysis in the single-center derivation cohort yielded an AVA_CTA_ threshold of 0.96 cm² for optimal identification of severe AS compared to TOE-based classification, and 0.95 cm² compared to TTE. These findings were retrospectively tested in a second, multicenter validation cohort. Here, the derived AVA_CTA_ thresholds demonstrated good diagnostic performance (AUC 0.817 vs. TOE; AUC 0.698 vs. TTE). Using the single-center derived, TOE-based safety thresholds of 1.17 cm² (to exclude) and 0.95 cm² (to confirm severe AS) yielded a sensitivity of 97.3% and specificity of 72.6% in the validation cohort.

AVC was identified as an independent marker of AS severity, as it showed poor correlation with AVA_TTE_, AVA_TOE_, AVA_CTA_, and AV p_mean_ in both cohorts. Among all modalities, AVA_TOE_ and AVA_CTA_ exhibited the strongest correlation (*r* = 0.73 in the derivation and *r* = 0.60 in the validation cohort), further highlighting that both parameters reflect the anatomic AVA.

Compared to prior studies reporting CT-based thresholds between 1.2 and 1.3 cm² [18–22], our optimal threshold of 0.96 cm² was lower. Methodological differences in CT image acquisition and planimetry, including potentially more conservative and tighter measurement techniques, along with specific patient cohort characteristics - such as a higher prevalence of low-gradient AS and preserved ejection fraction - may contribute to this discrepancy. While 3D-echocardiographic techniques and image fusion models have been proposed to improve planimetry and account for the non-circular geometry of the left ventricular outflow tract (LVOT), these methods are not yet widely used in clinical practice [20, 23–26]. A recent smaller retrospective study reported poor diagnostic performance of AVA_CTA_ in discriminating AS severity when using AVC as a reference standard [19]. However, AVC does not account for flow status or valve fibrosis - both critical contributors to AS pathophysiology - as noted by the study authors. Furthermore, the distribution of calcium (e.g., leaflet tip vs. peripheral calcification) substantially affects its hemodynamic and likely clinical relevance. Severe AS can occur in the absence of significant calcification, particularly in younger patients, women, and those with bicuspid valves - populations that are often underrepresented and prone to misclassification when relying solely on AVC as noted by the authors [19].

**Fig. 5:**
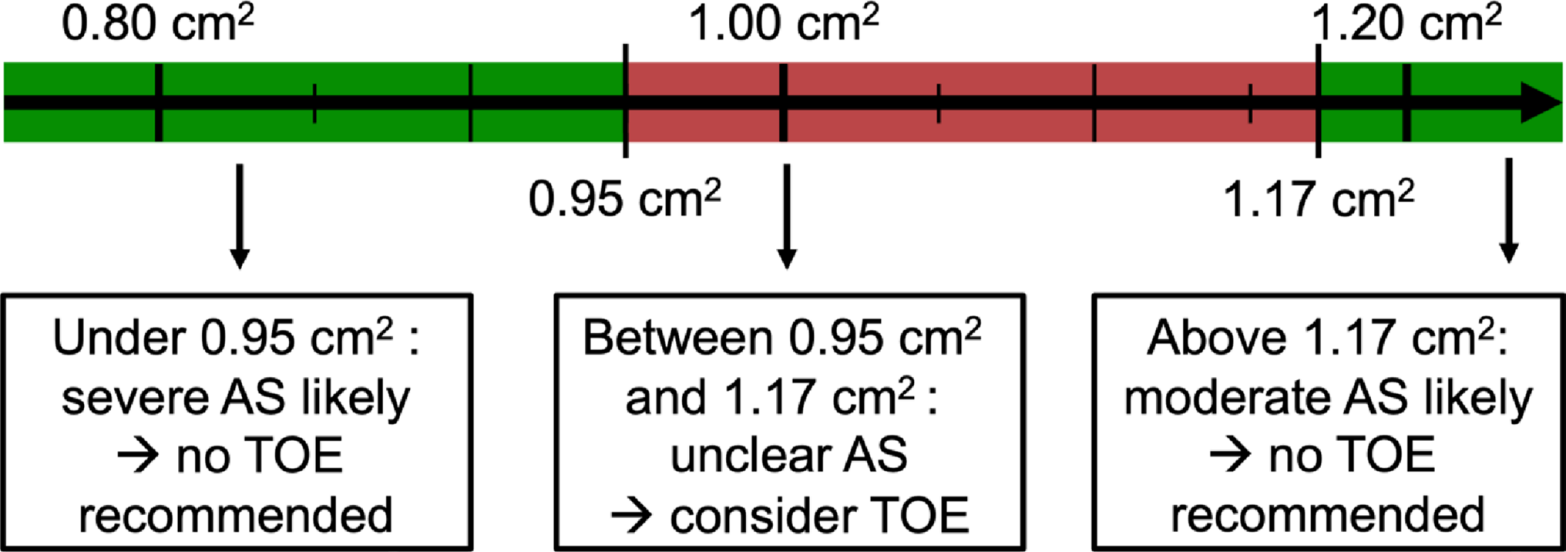
Threshold recommendations for clinical decision-making based on AVA_CTA_ values derived from ROC analysis using AVA_TOE_ as reference. AVA_CTA_ < 0.95 cm² suggests severe aortic stenosis; AVA_CTA_ >1.17 cm² implies moderate aortic stenosis. For values between 0.95 cm² and 1.17 cm², additional evaluation with TOE is recommended

Despite known modality-specific discrepancies in AVA measurements, no established cut-off values are currently defined for AVA_CTA_ [5, 8, 18–20]. Based on our findings, we propose an AVA_CTA_ threshold of 0.96 cm² for defining severe AS. During TAVR work-up, CTA can therefore reliably confirm or exclude severe AS using the proposed modality-specific safety thresholds (0.95 cm² and 1.17 cm²). In cases with AVA_CTA_ outside this grey-zone additional TOE planimetry is certainly dispensable. These recommendations for TOE performance based on AVA_CTA_ measurement are illustrated in Fig. 5.

### Limitations

This study has several limitations. Due to the retrospective study design, there is a selection bias towards borderline cases in whom all three imaging modalities were available. Moreover, patients with moderate AS and women were underrepresented in both cohorts, despite a relatively high proportion overall, and only symptomatic patients were included, which limits the generalizability of the results. TTE and TOE pose various challenges, including measurement of the LVOT diameter and quantification of LVOT stroke volume. Performing AVA planimetry can be difficult in the presence of severe calcification in both TOE and CTA acquisitions. However, all examinations were conducted and interpreted in accordance with international guidelines. AVA_TOE_, when performed by the same investigator following TTE, may not have been blinded and could have introduced bias towards the TTE results. Additionally, no further diagnostic procedures, such as dobutamine stress echocardiography or invasive catheterization, were used to exclude pseudo-severe AS in patients with low-flow subtypes.

## Conclusion

This is the first study to evaluate a comprehensive multimodal TAVR work-up that includes AVC, AVA_TTE_, AVA_TOE_, and AVA_CTA_, with the aim of defining AVA_CTA_-based thresholds for aortic stenosis severity and validating these thresholds in an independent multicenter enrolled validation cohort. AVA_CTA_ demonstrated strong correlation with AVA_TOE_ and a good diagnostic performance, with a slightly lower optimal cut-off (approx. 0.96 cm²) for severe AS. As such, AVA_CTA_ represents a valuable and reliable parameter in the multiparametric assessment of AS.

## Supplementary Information

Below is the link to the electronic supplementary material.

Supplementary Material 1

## Data Availability

The data underlying this article are available from the corresponding author upon reasonable request.

## Abbreviations

AS: aortic stenosis.
AUC: area under curve.
AV: aortic valve.
AVA: aortic valve area.
AVA_CTA_: aortic valve area measured by computed tomography planimetry.
AVA_TOE_: aortic valve area measured by transesophageal echocardiography planimetry.
AVA_TTE_: aortic valve area calculated by continuity equation on echocardiography.
AVC: aortic valve calcification.
AV p_mean_: mean trans aortic valve pressure gradient.
CAD: coronary artery disease.
CT: computed tomography.
CTA: computed tomography angiography.
DRG: diagnosis related groups.
IRR: interrater reliability.
LVEF: left ventricular ejection fraction.
LF/LG: low flow, low gradient.
ROC: receiver operator characteristic curve.
SD: standard deviation.
SVi: stroke volume index.
TAVR: transcatheter aortic valve replacement.
TOE: transesophageal echocardiography.
TTE: transthoracic echocardiography.
