# Supplementary material for: Multimodality assessment of aortic valve area in aortic stenosis: a multicenter validation study
Source: Int J Cardiovasc Imaging. 2025 Dec 1;42(1):99–109. doi: 10.1007/s10554-025-03576-7 (PMC12847234; doi:10.1007/s10554-025-03576-7)
Supplement: Supplementary file 1 — Supplementary Material 1 [file 10554_2025_3576_MOESM1_ESM.docx]

**Supplement Table 1: Subgroup characteristics of the all patients:**

|  | **Concordant high gradient AS** | **Disconcordant high gradient AS** | **LF/LG AS with reduced EF** | **LF/LG AS with preserved EF** | **Normal flow, low gradient AS** | **Moderate AS** |
| --- | --- | --- | --- | --- | --- | --- |
| N | 126 (21.6) | 25 (4.3) | 92 (15.8) | 86 (14.8) | 130 (22.3) | 124 (21.3) |
| Age, years | 80.4 ± 7.9 | 78.9 ± 5.3 | 80.7 ± 7.5 | 82.1 ± 6.8 | 80.0 ± 6.2 | 80.8 ± 6.9 |
| **Gender**  Male, n (%)  Female, n (%) | 60 (47.6)  66 (52.4) | 20 (80.0)  5 (20.0) | 54 (58.7)  38 (41.3) | 32 (37.2)  54 (62.8) | 65 (50.0)  65 (50.0) | 77 (62.1)  47 (37.9) |
| **Body measurements**  Height, cm  Weight, kg  BMI, kg/m^2^ | 167.5 ± 9.1  77.4 ± 16.0  27.3 ± 4.3 | 171.4 ± 5.7  87.4 ± 18.7  29.7 ± 6.2 | 168.1 ± 9.0  75.8 ± 16.1  26.8 ± 5.2 | 166.2 ± 9.1  74.4 ± 15.6  26.8 ± 5.0 | 166.6 ± 9.6  73.2 ± 14.4  26.2 ± 4.2 | 170.1 ± 8.4  79.2 ± 15.4  27.1 ± 4.6 |
| **TTE findings**  LVEF, %  > 50, n (%)  30-50, n (%)  < 30, n (%)  AV p_mean_, mmHg  AVA_TTE_, cm^2^  Diastolic dysfunction  Stroke volume, ml/m^2^ | 53.2 ± 8.9  98 (77.8)  24 (19.0)  4 (3.2)  50.2 ± 9.9  0.68 ± 0.16  51 (40.5)  40.6 ± 11.2 | 57.2 ± 6.5  23 (92.0)  2 (8.0)  0 (0.0)  48.1 ± 5.5  1.14 ± 0.15  6 (24.0)  55.9 ± 14.5 | 37.8 ± 8.4  0 (0.0)  68 (73.9)  24 (26.1)  24.6 ± 7.8  0.73 ± 0.15  36 (39.1)  25.9 ± 5.3 | 56.9 ± 4.7  86 (100.0)  0 (0.0)  0 (0.0)  26.7 ± 7.2  0.75 ± 0.12  22 (25.6)  29.5 ± 4.7 | 52.2 ± 8.6  99 (76.2)  27 (20.8)  4 (3.1)  30.9 ± 7.8  0.83 ± 0.14  51 (39.2)  43.4 ± 7.5 | 47.6 ± 12.2  72 (58.1)  39 (31.4)  13 (10.5)  26.2 ± 7.9  1.12 ± 0.14  41 (33.1)  40.6 ± 10.8 |
| **TOE findings**  AVA_TOE_, cm^2^ | 0.70 ± 0.22 | 0.98 ± 0.19 | 0.81 ± 0.23 | 0.81 ± 0.18 | 0.87 ± 0.22 | 1.00 ± 0.20 |
| **CT findings**  AV calcium score, AU  AVA_CTA_, cm^2^ | 2607.0 ± 2176.4  0.78 ± 0.19 | 4878.0 ± 3440.4  1.00 ± 0.22 | 2253.4 ± 1701.9  0.86 ± 0.21 | 1913.1 ± 1169.1  0.86 ± 0.15 | 1995.0 ± 1575.7  0.93 ± 0.22 | 2046.9 ± 1732.0  1.04 ± 0.22 |
| **Preexisting illnesses**  Diabetes mellitus, n (%)  Hypertension, n (%)  Nicotine abuse, n (%)  CAD, n (%)  Atrial fibrillation, n (%) | 44 (34.9)  107 (84.9)  24 (19.1)  67 (53.2)  59 (46.8) | 10 (40.0)  17 (68.0)  7 (28.0)  19 (76.0)  4 (16.0) | 39 (42.4)  75 (81.5)  18 (19.6)  70 (76.1)  34 (37.0) | 29 (33.7)  72 (83.7)  11 (12.8)  51 (59.3)  34 (39.5) | 40 (30.8)  107 (82.3)  25 (19.2)  82 (63.1)  51 (39.2) | 37 (29.8)  102 (82.3)  30 (24.2)  76 (61.3)  44 (35.5) |

**Supplement Table 1:** Subgroup characteristics of all patients (n = 583). Values are presented as frequency (%) for categorical variables or mean ± SD for continuous variables. Abbreviations: AS, aortic stenosis; LF/LG, low flow, low gradient; BMI, body mass index; LVEF, left ventricular ejection fraction; AV p_mean_, aortic valve mean pressure gradient; AVA, aortic valve area; AVA_TTE_, AVA calculated by the continuity equation in transthoracic echocardiography; AVA_TOE_, AVA measured by transesophageal echocardiography planimetry; AVA_CTA_, AVA measured by computed tomography planimetry; CAD, coronary artery disease; SD, standard deviation.
